# Advancements in Aerogel Technology for Antimicrobial Therapy: A Review

**DOI:** 10.3390/nano14131110

**Published:** 2024-06-28

**Authors:** George-Alexandru Croitoru, Diana-Cristina Pîrvulescu, Adelina-Gabriela Niculescu, Marius Rădulescu, Alexandru Mihai Grumezescu, Carmen-Larisa Nicolae

**Affiliations:** 1 Faculty of Dental Medicine, Carol Davila University of Medicine and Pharmacy, 050474 Bucharest, Romania; alex.croitoru@umfcd.ro (G.-A.C.); carmen-larisa.nicolae@umfcd.ro (C.-L.N.); 2 Faculty of Chemical Engineering and Biotechnology, National University of Science and Technology Politehnica Bucharest, 011061 Bucharest, Romania; diana.pirvulescu@stud.fim.upb.ro (D.-C.P.); adelina.niculescu@upb.ro (A.-G.N.); agrumezescu@upb.ro (A.M.G.); 3 Research Institute of the University of Bucharest—ICUB, University of Bucharest, 050657 Bucharest, Romania

**Keywords:** aerogels, antimicrobial resistance, antimicrobial therapy, drug delivery, antimicrobial applications, aerogel-based coatings

## Abstract

This paper explores the latest advancements in aerogel technology for antimicrobial therapy, revealing their interesting capacity that could improve the current medical approaches for antimicrobial treatments. Aerogels are attractive matrices because they can have an antimicrobial effect on their own, but they can also provide efficient delivery of antimicrobial compounds. Their interesting properties, such as high porosity, ultra-lightweight, and large surface area, make them suitable for such applications. The fundamentals of aerogels and mechanisms of action are discussed. The paper also highlights aerogels’ importance in addressing current pressing challenges related to infection management, like the limited drug delivery alternatives and growing resistance to antimicrobial agents. It also covers the potential applications of aerogels in antimicrobial therapy and their possible limitations.

## 1. Introduction

Antimicrobial resistance (AMR) is a worldwide concern that requires research. It represents the ability of microorganisms, including bacteria, viruses, fungi, and parasites, to resist the effects of antimicrobial agents, which are drugs used to treat infections. Antimicrobial medications become ineffective due to bacteria’s ability to evolve and adapt to them. It compromises the ability to prevent and cure a growing number of illnesses effectively. AMR is one of the significant dangers to world health in the 21st century, according to the World Health Organisation (WHO) [1,2]. AMR has been found to be caused mainly by the overuse of antibiotics. The effects of antibiotic resistance are especially dangerous for vulnerable patients undergoing joint replacement, dialysis, chemotherapy, and surgery. These people are more prone to infections because they frequently have compromised immune systems due to their illnesses or treatments. Longer hospital stays and more expensive medical care can result from it. Furthermore, antibiotic resistance significantly affects people who have long-term health issues like diabetes, asthma, and rheumatoid arthritis [3].

Antimicrobial-resistant illnesses have a significant impact on mortality, and the seriousness of the problem was shown by a 2022 study that found antimicrobial-resistant infections to be third in line for causes of death, following cardiovascular disorders [4]. Furthermore, almost 5 million deaths were linked in some way to drug-resistant illnesses, demonstrating the extensive effects of AMR. Antimicrobial-resistant diseases are predicted to cause 10 million fatalities annually by 2050, which is a concerning prediction for the future. The fact that this prediction exceeds the anticipated death toll from cancer indicates how urgently antibiotic resistance needs to be addressed [4,5].

Several factors contribute to the advancement of AMR, such as antibiotic overuse in human treatments, overuse in agriculture and animals, lack of education, and many others. Although regulatory legislation has been made in both the US and the European Union to limit the use of prophylactic antibiotics in animals, there are still cases where it is allowed. Preventive use of antibiotics in single animals and small groups is allowed under veterinary surveillance, which still contributes to AMR [6].

Many people believe that viral infections like the flu or common cold may be successfully treated with antibacterial drugs like antibiotics. Because viral infections do not respond to antibiotic treatment, this misperception leads to the inappropriate prescription of antibiotics for these diseases [3]. Prescription medicine, especially antibiotics, is a major aspect of patient care in many underdeveloped countries where access to adequate diagnostic tools may be limited. Antibiotic misuse and overuse result from this dependence on antibiotics in the absence of a proper diagnosis, which fuels the development and spread of AMR [3].

Antibiotic-resistant bacteria are given the opportunity to survive when antibiotics are used in agriculture, which speeds up the emergence of AMR. Public health is at risk because these resistant bacteria can infect people through direct contact, contaminated food, and the environment. In many cases, antibiotics are added to the water and food given to the animals as a preventative measure for diseases, contributing to AMR in time. The issue can be made worse by resistant microorganisms and drug residues that can linger in the environment [7].

There is a need for continuous research to overcome AMR. Misconceptions about antibiotics, inadequate diagnostic tools, and poor-quality medications further exacerbate the issue. Therefore, urgent action is needed to address AMR through coordinated efforts, including responsible antibiotic use, enhanced surveillance, improved diagnostics, strengthened regulations, and novel treatments that exclude antibiotics [8]. Specifically, this review aims to discuss the efficacy of aerogels as antimicrobial agents by examining their unique properties and potential applications in medicine.

Aerogels are a class of nanoporous materials that have unique characteristics. They have a high surface area, low density, and superior thermal insulation qualities. Aerogels are very lightweight and originate from molecular precursors that are hybrid, inorganic, or organic. Air makes around 80–99.8% of the structure of aerogels. Consequently, they are gel-like solids with empty spaces in their structure, making them ultra-lightweight [9]. Aerogels’ unique porous structure initially allows for quick water intake and works effectively as a carrier for bioactive substances. When necessary, this structure’s high loading capacity for drugs ensures improved stability during storage and facilitates medication release. Because the regulated and effective release of bioactive chemicals is crucial for the effectiveness of treatment in drug delivery systems, aerogels have a promising future for such applications [9,10].

In this context, aerogels exhibit promising potential for innovating antimicrobial therapies and overcoming AMR. Thus, this paper aims to shed some light on the advances in aerogel technology, emphasizing their utility for better-performing treatment strategies. To create a comprehensive view of aerogels, the following sections classify these versatile structures, describe their properties, detail the mechanisms of antimicrobial activity, and present the methods to synthesize and modify them for antimicrobial use. Further, the applications of aerogels in antimicrobial therapy are discussed, highlighting their roles in wound healing and dressings, drug delivery, and biomedical coatings.

## 2. Aerogels Classification

Different types of aerogels have been synthesized since they were first discovered in 1931 by S.S. Kistler [11]. The classification of aerogels is presented in Table 1.

Over the years, one of the most widely used was silica aerogels because of their great properties. They have low thermal conductivity, usually between 12 and 20 mW/mK [13]. Their extremely low density, which ranges from 0.003 to 0.5 g/cm^3^ [14], ensures they are suitable for applications where weight is a concern. Furthermore, they have a wide surface area due to their porous structure, which allows them to interact with other substances more easily. This makes them useful for drug administration, catalysis, and adsorption applications. However, the application range of silica-based aerogels is limited due to their fragility, poor mechanical characteristics, and need for a time-consuming processing method. Therefore, the synthesis method and its structure need to be improved, as well as the use of other materials in aerogels production [15,16].

Significant characteristics emerged across the range of aerogel types, such as silica-based, polymer-based, and hybrid forms, emphasizing their biodegradability, biocompatibility, permeability, and capacity to mimic biological structures. Aerogels are used in various biomedical applications, including implantable devices, biosensing, wound healing, regenerative medicine, drug delivery, and diagnostics [17].

Aerogel’s application in antimicrobial therapy is a new and promising area of medical research. Aerogels have many benefits for antimicrobial applications. They are well-known for their distinctive qualities, which include large surface area, porosity, and tunable features. These characteristics allow aerogels to be used as efficient antimicrobial agent carriers, allowing for targeted delivery and controlled release at infection sites. Furthermore, because of their biocompatibility, biodegradability, and functionalization potential, they are excellent choices for treating microbial infections with minimal negative effects. Aerogels have the potential to improve treatment outcomes, lower drug resistance, and address recurring issues in antimicrobial therapy because of their capacity to encapsulate and protect antimicrobial compounds [18,19].

As antimicrobial aerogels are extensively being studied, numerous creations are patented to secure their intellectual property and drive commercial development [20]. For aerogels used in antimicrobial applications, patents provide an important source of data and can be used as an inspiration for future studies [21]. Patent CN111529748B describes and protects a composite dressing for wound repair, made of three layers and based on chitosan/polyvinyl alcohol/oxidized sodium alginate. The composite material is breathable, has high porosity, and can absorb wound exudate and keep the wound clean [22]. Patent CN103520767B focuses on an aerogel dressing, mainly composed of water-miscible high molecular polymer, for wound healing with antibacterial properties. The in vitro experiments proved its nontoxicity and antibacterial effect, killing over 70% of the bacteria [23]. Patent CN118021521A covers and protects a multilayer antibacterial gauze based on friction nano power generation. The invention has an inner and outer gauze layer, with an antibacterial layer containing nano-sized ZnO particles. When these ZnO particles are exposed to UV radiation, they create oxidative free radicals. These radicals are very reactive and can destroy bacteria and viruses through oxidation. Therefore, this invention can be used for wound healing since it has antimicrobial properties [24]. Patent CN117918378A involves a silver-based aerogel with antibacterial properties. This invention can be used as a coating for materials or to improve the antibacterial effects of other materials. The method of preparation is also described [25].

Generally, the use of naturally occurring, biocompatible, and biodegradable materials when producing aerogels is preferred. Natural polymers are a great choice due to their properties. Conventional materials—silica, carbon, metals, and synthetic polymers, which are used to fabricate aerogels—have specific drawbacks that limit their applicability in biomedical applications. Among these disadvantages is the possibility of the body rejecting the material through the immune system and its cytotoxicity, which may cause unfavorable immunogenic reactions [26]. Several types of natural polymers that can be used as precursors for aerogels and their advantages are presented in Table 2.

### 2.1. Chitosan-Based Aerogels

One of the most prevalent biopolymers in the world is chitosan. This linear polysaccharide, produced from chitin through the deacetylation process, is well-known for its unique attributes, including mucoadhesiveness, antibacterial and anti-inflammatory activity, biocompatibility, and biodegradability. Scientists are becoming more and more interested in the process of creating aerogels from chitosan. The resulting aerogels’ unique characteristics, including their large surface area, mechanical strength, high degree of polymerization, and purity, make them intriguing for a wide range of uses [31]. Chitosan aerogel has antibacterial qualities that arise from its ability to rupture the cell membranes of microorganisms such as fungi and bacteria. Due to membrane failure brought on by this disruption, chitosan is able to enter cells and disrupt intracellular components. Furthermore, chitosan modifies the cell membrane’s permeability, which decreases the survival of the microbes even more [15,31]. Studies have demonstrated the efficacy of chitosan-based aerogels, like one conducted by Lin et al., where the antimicrobial study of chitosan aerogels suggested a strong antibacterial activity against Gram-negative and Gram-positive pathogens [32].

### 2.2. Alginate-Based Aerogels

Alginate is a naturally occurring polysaccharide that is isolated from seaweed. It is efficient against various bacteria and fungi due to its inherent antibacterial properties. Its safety for usage in biomedical applications is ensured by its biocompatibility, biodegradability, and nontoxicity [33,34]. Alginate facilitates the development of hydrogels because, in mild conditions, it gels via Ca(II)-mediated ionotropic gelation. Ultimately, different manufacturing techniques, such as freeze-drying, provide an environmentally friendly way to create alginate-based aerogels from wet gels while maintaining the crosslinked three-dimensional structures. In addition, the produced aerogels accelerate hemostasis by achieving the continuous release of Ca(II), a blood coagulation factor. Alginate’s capacity to rupture microbial cell membranes and obstruct their metabolic functions gives rise to its antibacterial qualities, which in turn cause microbial suppression or death [35,36].

### 2.3. Cellulose-Based Aerogels

Cellulose has drawn particular interest because of its great mechanical strength, hydrophilicity, biocompatibility, nontoxicity, and chemical and thermal stability. Aerogels can be made from cellulose from various sources, including plants, bacterial cellulose (BC), and conventional vegetable cellulose from wood. Because of its distinct structure from plant cellulose, BC has unique qualities that make it stand out. Because microorganisms synthesize BC in a chemically pure form, it has finer fibrils, typically between 20 and 80 nanometers in thickness, although they have the same chemical composition. The mechanical strength and purity of BC are enhanced by this finer structure. Moreover, BC is more stable than plant cellulose because of its greater degree of crystallinity [37,38,39].

Since cellulose has no direct antimicrobial effect, it can be easily loaded with antimicrobial agents, such as silver nanoparticles or other substances [40]. A study conducted by Shan Ye et al. proved that cellulose aerogels loaded with amoxicillin demonstrated antibacterial activity against *E. coli*, *C. albicans*, *S. aureus*, and *B. subtilis* [41].

## 3. Fundamentals of Aerogels

### 3.1. Structure and Properties

**Figure 1:**
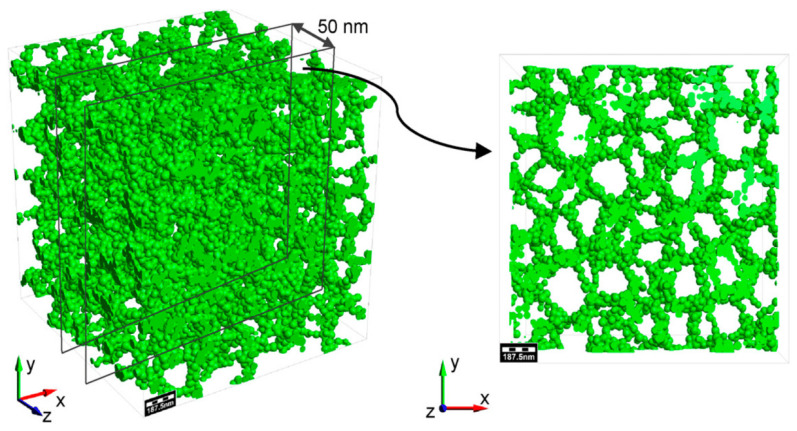
Aerogel’s general structure and its local structure. The green part represents clusters of atoms or nanoparticles forming the aerogel’s network. The spaces between them represent the pores. Reprinted from an open-access source [44].

Aerogels are highly porous materials, with the size of the pore being within the nanometer scale, which is interconnected. The aerogel’s structure is illustrated in Figure 1. Densities normally range from 0.001 to 0.5 g/cm^3^, and they are among the lightest solid materials. The porosity contributes to their low density and high surface area, typically ranging from several hundred to several thousand m^2^/g. Because of their high surface area, the adsorption and binding of antimicrobial agents onto the aerogel structure are improved, increasing their effectiveness against microbial pathogens [42,43].

Aerogels also possess thermal conductivity, the process by which heat is transferred through hollow and aerogel fibers. It is measured using Fourier’s law’s fundamental idea and expressed in W/(m·K). When it comes to thermal conduction, air has the lowest value among solid structures, and since aerogels have a porosity of nearly 99%, this lowers the conductivity of heat. Even if it is not usually the leading factor taken into account when using aerogels for antimicrobial purposes, this is still an important aspect to consider. Thermal conductivity may not be crucial in antimicrobial applications, but it has a secondary role, especially when they involve biomedical environments like implants or wound dressings. For instance, aerogels possessing low thermal conductivity can aid in keeping a consistent temperature surrounding the wound site, facilitating ideal healing environments in wound healing applications. Aerogels with low thermal conductivity can also minimize heat transfer to surrounding tissues in implanted devices or prosthetics, lowering the risk of thermal damage or patient discomfort [42,45]. The porosity, pore size, density, and consequent surface area depend on aerogels’ synthesis method and precursors [46].

### 3.2. Biocompatibility and Toxicity Considerations

The degree of biocompatibility exhibited by aerogels might vary based on their composition, manufacturing method, and intended use. Choosing an appropriate aerogel composition makes achieving biocompatibility simple. Biomedical applications such as tissue engineering, drug administration, and wound healing can benefit from the naturally derived and biocompatible nature of different aerogel materials, for example, cellulose and alginate [47,48].

Even though many aerogel materials are biocompatible, it is important to consider any possible toxicity concerns, particularly in medical applications where aerogels can come into contact with blood or living tissues. Certain aerogel materials, especially those functionalized with nanoparticles or synthetic polymers, can cause immune system responses or release toxic chemicals [49,50]. However, in most of the studies that address the toxicity of aerogels, the results show low to no toxicity at all. For instance, Mohammadian M. et al. wanted to address the toxicity of aerogels due to the limited available data for this. They made silica aerogels in combination with Ketoprofen and evaluated the cell viability through an MTT (3-[4,5-dimethylthiazol-2-yl]-2,5 diphenyl tetrazolium bromide) assay. The silica aerogels turned out to have no cytotoxicity and great potential for cell growth and survival [51]. In another study by Batista M. et al., the researchers synthesized chitosan-alginate aerogels with different concentrations. All eight aerogels examined demonstrated a non-cytotoxic effect, none having cytotoxicity percentages below the standard threshold of 70%, according to ISO 10993-5 [52].

In a study conducted by M. Alsmadi et al., the researchers synthesized chitosan-alginate aerogels loaded with cisplatin for lung cancer treatment. Although the aerogel showed a controlled release of the drug and reduced the mortality rate of the rat models, it also showed increased hepatic toxicity and mild renal toxicity, which was correlated with the administered dose [53]. Another study showed the chitosan aerogel’s potential biocompatibility for medical applications, caused by its low cytotoxicity in an epithelial colon cell line (HT29) used for wound dressing. A feature of chitosan-based materials, the scaffold’s positive charge, is probably responsible for its biocompatibility. The positively charged amino groups found in chitosan interact with negatively charged cell membranes to promote cell adhesion and proliferation. The disruption of bacterial cell membranes and inhibition of microbial growth further add to the aerogel’s antimicrobial properties [54].

However, more studies indicate that inorganic and carbon-based aerogels are more toxic than polymeric ones. Aerogels based on carbon, such as graphene, fullerenes, and carbon nanotubes (CNTs), have attracted much attention because of their special qualities, including great mechanical strength, electrical conductivity, and large surface area. They were limited, nevertheless, by their possible toxicity, low biodegradability, poor solubility in fluid environments, and tendency to accumulate. Studies have demonstrated that whereas low concentrations of reduced graphene oxide (rGO) are usually safe in animal models, higher dosages can cause serious toxicity, especially in organs, including the kidneys, liver, and lungs. Low dosages of rGO (<1 mg/kg) in rat experiments were shown to be safe, with no visible negative effects. Yet, rGO demonstrated a dose-dependent increase in toxicity at higher dosages (1–10 mg/kg), which had negative effects on the kidneys, liver, and lungs. Because rGO mainly accumulates in these organs, where it can cause tissue damage, oxidative stress, and inflammation, its toxicity is evident [55,56].

Overall, studies show that most aerogels produced from natural polymeric materials demonstrate low or no toxicity at all, with a few exceptions. These exceptions mainly happen because of the high or repetitive dosage, which can be adjusted to achieve a lower toxicity.

## 4. Mechanisms of Antimicrobial Activity

One of the aerogel’s mechanisms of antimicrobial activity is its capacity to swell. Aerogels promote the retention of a moist wound environment, which is essential for facilitating the migration and proliferation of different cell types involved in the healing process. Aerogel’s capacity to absorb excess moisture from the wound site helps prevent fluids from affecting the surrounding healthy tissue, which lowers the chance of recurrent infections by creating an inhospitable habitat for microbial proliferation and accelerates the healing process. Furthermore, aerogels are a promising option for controlling hemorrhage. They can rapidly absorb blood from a bleeding wound, which helps form a clot and reduce blood loss, creating a hemostatic barrier [36,57,58,59].

**Figure 2:**
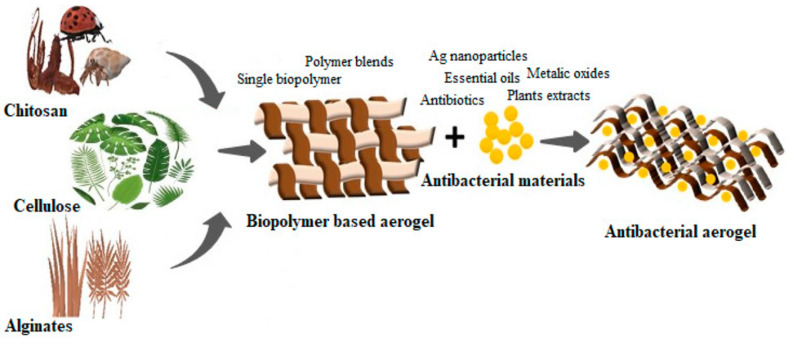
The loading of aerogels with antimicrobial agents. Reprinted from an open-access source [60].

As mentioned before, aerogels can have antimicrobial effects, depending on the chosen material. Chitosan, cellulose, and alginate are excellent choices because they hold these properties. Moreover, drugs or other antimicrobial agents, like metal nanoparticles, essential oils, and enzymes, can be loaded into the aerogel, as presented in Figure 2 [60].

The rate and method of the substance or drug release from aerogels in aqueous conditions are determined by multiple important factors:

(a) Erosion and degradation of the carrier matrix: Encapsulated drug molecules may be released as a result of the aerogel matrix’s slow breakdown or deterioration. Factors like the aerogel material’s stability and composition may have an impact on this process.
(b) Degree of the drug molecules’ interaction with the carrier’s structure: The drug molecules’ release kinetics are influenced by their affinity for the aerogel matrix. While weaker interactions may lead to faster release, stronger interactions may result in slower release rates.
(c) Drug and aerogel hydration: Water leaking into the aerogel matrix can cause swelling and drug hydration in both the encapsulated drug and the carrier. This hydration process can influence the drug molecules’ ability to diffuse out of the aerogel structure [61].

Many studies have proved the antimicrobial effect of aerogels. Chitosan/mesoporous silica hybrid aerogel demonstrated antibacterial effects against *S. aureus* and *E. coli*, where it reduced about half of the bacteria. Chlorinated Chitosan/mesoporous silica hybrid aerogel demonstrated about 100% bacteria inactivation within 30 and 10 min, respectively, showing the system’s strong antibacterial potential [62]. In a study by Khan et al., silver nanoparticles (AgNPs) and enzymes were used as antibacterial materials and bound within cellulose nanofiber (CNF) aerogels. The study’s findings demonstrated the aerogels’ non-toxic and biodegradable characteristics, proving their safety and applicability in drug delivery. It was efficient at protecting the loaded agents’ enzymatic and antibacterial properties when cellulose nanofibers were used as a support matrix for bioactive compounds. This shows that CNF aerogels may be a useful tool for the delivery of bioactive substances. Moreover, cellulose nanofibers’ biocompatible and biodegradable characteristics improve the aerogel composites’ overall safety profile. All things considered, this can be explained by the combined antibacterial action of the AgNPs and the cationic CNF. Silver’s antibacterial effect is caused by its ability to attach to negatively charged bacterial cell walls. This results in the interruption of cellular respiration and cell wall permeability, which causes the disintegration and death of the bacteria [63].

In another study, ampicillin was loaded into chitosan aerogels, where it exhibited a strong antimicrobial effect. The ampicillin and chitosan worked in conjunction to increase the efficacy against various bacteria. Even at lower ampicillin dosages, this synergistic impact helped produce the strong antibacterial activity observed. Additionally, in vitro cytotoxicity testing on human cells proved the aerogel composites’ biocompatibility. Furthermore, the chitosan aerogels were shown to effectively speed wound healing in an in vivo rat wound model, demonstrating their potential for antimicrobial treatments and wound healing [64].

Studies have demonstrated that when metallic nanoparticles, such as silver or copper, are incorporated into aerogels, they offer a potent antimicrobial defense mechanism through the generation of reactive oxygen species (ROS). The porous matrix of aerogels makes it simpler for the metallic NPs to disperse and stick throughout the aerogel structure. This ensures a prolonged antibacterial effect and a sustained release of metallic nanoparticles. Aerogels’ large surface area also makes it possible for nanoparticles to be exposed to light or ambient conditions effectively, which generates ROS. These target vital biological constituents like DNA, proteins, and lipids, damaging the microbial cells. ROS cause oxidative damage, which leads to microbial cell death by disrupting essential cellular functions and weakening microbial structures [65,66,67].

Photocatalytic aerogels represent a novel approach to antimicrobial technology by combining aerogels and photocatalytic materials like TiO_2_. Their mechanism of action involves using light as a catalyst to create chemical processes that produce ROS. TiO_2_ present in the porous structure of aerogels begins photocatalysis when exposed to UV light, resulting in the production of ROS, such as e superoxide ions and hydroxyl radicals. The combination of aerogel’s high surface area and photocatalytic activity amplifies its antimicrobial efficacy [68,69].

## 5. Synthesis and Modification of Aerogels for Antimicrobial Use

### 5.1. Synthesis Techniques

The properties of the precursors, as well as the conditions for the gelation processes, have an essential impact on the structure and properties of aerogels. The main methods for creating gels are sol-gel processes, which can be generally divided into two categories. One method uses hydrolysis to start polycondensation processes, which dissolve a monomer in a solvent and create a gel network. This method works especially well for creating aerogels from inorganic precursors or metal oxides [70].

The alternative method is dissolving a polymer in a solution, followed by chemical or physical crosslinking to reassemble the polymer network (e.g., changing the pH or temperature). This technique is frequently used to create aerogels from biopolymers, such as cellulose or chitosan [70].

**Figure 3:**
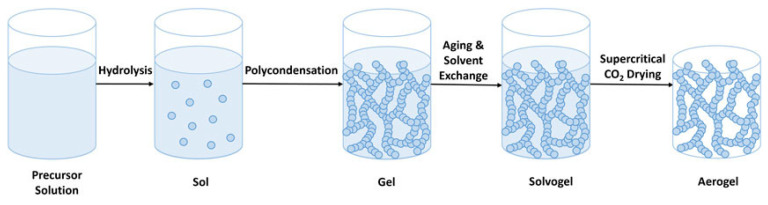
The general steps for aerogel synthesis through sol-gel. Reprinted from an open-access source [70].

The general synthesis process is presented in Figure 3. The aerogel synthesis usually starts with precursor solution preparation, which involves dissolving or dispersing the needed aerogel-forming components (such as metal oxides or polymers) in an appropriate solvent. Surfactants or other additives may also be included in the precursor solution to control gelation and alter the characteristics of the final aerogel. Then, the dispersed particles or polymer chains in the precursor solution undergo gelation. Afterward, they collect and crosslink to create a three-dimensional network that is referred to as a gel. Various techniques, including chemical reactions, temperature changes, and pH modifications, can be used to achieve gelation. The aging process strengthens the gel, which reduces shrinking during the drying phase. After that, a procedure known as solvent exchange or supercritical drying is used to extract the solvent from the gel. In solvent exchange, the initial solvent is replaced with a volatile one, like alcohol, by immersing the gel in a succession of solvents with increasingly decreasing surface tension. To extract the solvent from the gel while preserving its solid structure, supercritical drying involves exposing the gel to supercritical conditions (such as supercritical CO_2_) [70,71,72].

The drying process is one of the most important and prominent steps in the production of aerogels. In this process, the solvent is extracted from the gel structure while maintaining its porosity network, which leads to the formation of the solid aerogel material. Several drying techniques are used [71], including supercritical drying, lyophilization or freeze-drying, ambient pressure drying (subcritical drying), and evaporation [71].

Supercritical drying is defined as the solvent being extracted from the gel’s pores using supercritical fluids. When a fluid is compressed and heated above its critical temperature and pressure, it achieves its supercritical conditions. In these circumstances, the gel structure is prevented from collapsing, and its porous morphology is preserved because the liquid solvent passes straight from the liquid phase to the gas phase without first going through an intermediate liquid phase [73]. Various studies have proved the success of creating aerogels by using supercritical drying. Although many of them involve silica [72], others, like cellulose [74] or alginate [75], have created ultra-lightweight and highly porous aerogels. However, this method also has disadvantages, as it is highly complex and expensive.

Ambient pressure drying is an alternative method for drying aerogels that avoids the complexities and costs associated with supercritical drying. In this process, the gel is simply allowed to dry at ambient pressure and temperature conditions, typically in air or another inert atmosphere. The solvent progressively evaporates from the gel matrix, causing the porous structure to shrink slowly as the liquid is eliminated. This slow-drying procedure reduces the chance of collapse or damage while preserving the integrity of the gel structure. Aerogels made using ambient pressure drying may have somewhat lower porosity and surface area than those made with supercritical drying, but it is easier and less expensive. Furthermore, longer drying durations could be necessary to completely remove the solvent, particularly for denser or thicker aerogel samples [75,76].

### 5.2. Surface Functionalization and Modification

The aerogel’s surface can be modified to meet different needs. By doing so, it can improve the biocompatibility and enhance antimicrobial activity. A cellulose-based wet-stabilized aerogel with a porous structure that was able to remain intact even after being exposed to water was created in the study by Henschen et al. This durability raises the possibility of using the aerogel in a number of situations where water stability is required, for example, in wound dressings. Additionally, by successively adsorbing layers of poly(vinylamine) (PVAm) and poly(acrylic acid) (PAA), the researchers modified the aerogel’s surface. The charge and chemical composition of the aerogel’s surface may be precisely controlled by this multilayer coating method. Testing the aerogel’s capacity to adhere to bacteria demonstrated the importance of these surface modifications. Remarkably, the aerogel surface-coated displayed an impressive level of bacterial adhesion, as over 99% of bacteria adhered to its surface [77].

In another study, scientists synthesized lightweight nanoporous silica aerogels and then used a chemical method known as silane-based methylation to modify the surfaces of these materials to make them hydrophobic. Because of this surface alteration, the aerogels were water-repellent. They also discovered the ability of these modified aerogels to stop bacteria from adhering to their surfaces. This was particularly significant for two common bacteria: *S. aureus* and *E. coli*. The scientists effectively developed a barrier that prevented pathogens from attaching by modifying the aerogels’ surface to be hydrophobic. This alteration significantly reduced bacterial adherence and improved the aerogels’ capacity to repel water. There were two main causes for this antiadhesive behavior. First, germs were kept out of the small crevices and gaps on the surface by the air pockets present inside the aerogels’ porous structure. Second, the bacteria found it more difficult to adhere since the pores themselves reduced the bacteria’s attraction to the aerogel surface [78].

Overall, the results highlight how important surface modification is to improving aerogels’ antibacterial activity. Through modifications to their surface characteristics, such as adding particular functional groups or making them hydrophobic, scientists can significantly improve aerogels’ resistance to water absorption and inhibition of bacteria adhesion. The aerogels’ ability to fight bacterial infections is improved by this surface change, which also offers new opportunities for the creation of innovative materials with multifunctional characteristics.

### 5.3. Incorporation of Antimicrobial Agents

Incorporating antimicrobial agents into aerogels can be done through different methods, for example, impregnation, covalent bonding, encapsulation, surface modification, and electrospinning [79].

Supercritical drug impregnation technology has gained more attention because of its intrinsic qualities, which include low critical temperature, environmental friendliness, nontoxicity, and recyclability. This method is based on supercritical carbon dioxide (scCO_2_) fluids. In the process, the supercritical fluid fills the gaps in the aerogel’s porous network and pushes out any air or other gases that may be present. This makes it possible for the supercritical fluid to make close contact with the aerogel matrix, which helps the target material to be impregnated effectively [79].

In a study by C. Darpentigny et al., the researchers developed antimicrobial cellulose nanofibril aerogels using Thymol as an antimicrobial agent. Thymol was impregnated into the aerogel by using supercritical impregnation. The results showed a controlled release of the Thymol, and it demonstrated strong antibacterial action against one yeast and two bacteria [80].

In another study, scCO_2_ was used to impregnate mesoglycan (MSG), a therapeutic substance, onto a calcium alginate aerogel in order to create a material that can serve as a barrier to protect wounds and facilitate the healing process. The impregnation kinetics were investigated at 40 and 60 °C for 2–24 h contact durations. At both temperatures, equilibrium was established in 15 to 24 h, with higher temperatures enabling a higher loading of MSG onto the aerogel [81].

Covalent bonding is another method that can be used. To achieve covalent bonding, the surface of the aerogel is typically functionalized with reactive groups. The antimicrobial drugs, which have complementary functional groups, can react chemically with these groups. The strong attachment of the antimicrobial compounds to the aerogel matrix is ensured by the resulting covalent bonds, which eventually improve their retention and effectiveness [82].

Silica aerogel can be modified to create SA-COOH (a carboxylic group) to be used as drug carriers for CCB (Carvedilol). The two steps in this alteration method were grafting with 3-aminopropyltriethoxysilane (AEAPTES) and treating with succinic anhydride. The study is the first application of silica aerogel with carboxylic functionalization as a drug carrier. It was proven that the modification was successful, and the drug was loaded onto the aerogel. Further experiments showed that the system didn’t cause any cytotoxicity. This experiment opens new doors to study attaching antimicrobial agents to aerogels through covalent bonding [83].

## 6. Applications of Aerogels in Antimicrobial Therapy

**Figure 4:**
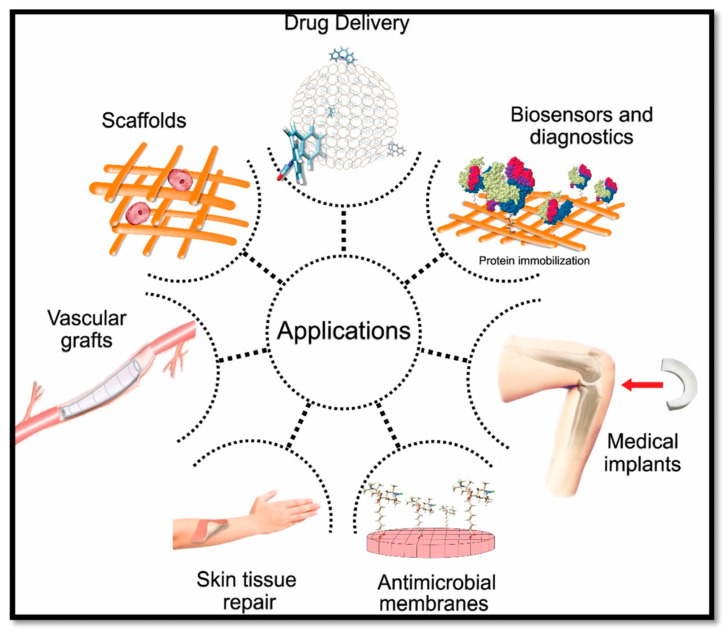
Biomedical applications of aerogels. Reprinted from an open-access source [85].

Aerogels have a lot of potential uses in antimicrobial therapy because of their properties, such as high porosity and large surface area. Aerogel matrices can be used in wound dressings, medical implants, medical devices, and drug delivery by adding antimicrobial agents to them [17,84]. The potential uses of aerogels in antimicrobial therapy are presented in Figure 4 [85].

Compared with conventional treatments or medicines, aerogels hold some additional advantages:

- Stability and controlled release: Aerogels can be loaded with drugs and promote a controlled release. This improves the drug’s stability and therapeutic effect, with a prolonged antimicrobial effect, compared to traditional drugs that may require repeated application [79,86].
- Reduced side effects: Through targeted therapy, aerogels can target only the desired pathogens without harming other healthy cells or tissues [87]. On the other hand, repeated administration and prolonged exposure to certain antibiotics can cause negative effects [88].
- Large surface area: The porous structure of aerogels offers a large surface area of interaction with microbes. This can improve the overall antimicrobial effect compared with traditional drugs, which usually lack pores [89,90].

The comparison between antimicrobial aerogels and traditional drugs and their potential effects is summarized in Table 3.

### 6.1. Aerogels in Wound Healing and Dressings

The body’s natural process of restoring injured tissue is called wound healing. It involves a number of intricate biological processes meant to repair the integrity of the skin and the underlying tissues. To promote the best possible healing, wounds must be correctly cared for, which may involve cleaning the area, applying wound dressings, or using topical medications [98].

Despite the wide range of materials used in wound dressings, aerogels have recently shown promise for this application. The ability to absorb fluids is extremely important for preventing bleeding because of the large volume of blood exudate that may leak from the wound. Aerogels are ideal for absorbing excess exudate from wounds, keeping a moist environment favorable to healing, and encouraging tissue regeneration. Furthermore, wound dressings must exhibit antimicrobial properties to prevent infections [99,100].

In a particular study, researchers developed a novel wound dressing using an antibiotic-free sodium alginate (SA) base incorporated with a photosensitizing agent called TPAPP and a crosslinking agent PBA (phenylboronic acid). This produced a SA@TPAPP@PBA aerogel, which was subsequently tested to see if it could effectively heal mice’s wounds. Antibacterial photodynamic therapy (aPDT) was used in the study as a therapeutic strategy. The SA@TPAPP@PBA aerogel was shown to be able to efficiently capture *S. aureus* through in vitro antibacterial testing. Moreover, the aerogel exhibited high porosity, which was beneficial for absorbing blood exudate from wounds and promoting rapid hemostasis [36]. The engineering of hybrid aerogel biomaterials that combine collagen and wheat grass nutraceuticals is a major development in the field of wound healing research. Collagen aerogels that have been functionalized with wheat grass bio-actives have improved mechanical, physicochemical, and porosity qualities that resemble the extracellular matrix observed in nature. With the help of this novel biomaterial, bioactive chemicals can be continuously delivered to the cellular environment, promoting angiogenesis and tissue regeneration [101]. Moreover, CNF aerogels with antibacterial incorporation improve wound care by preventing infections, supplying the ideal amount of oxygen permeability for cell growth, and inhibiting anaerobic bacteria growth [102]. Chitosan-cellulose aerogel microfibers loaded with ibuprofen demonstrated controlled release, biocompatible, and antibacterial against *E. Coli* and *S. aureus.* These results can be used in further developing materials for wound healing [103]. Another study evaluated the wound healing and antimicrobial activity of alginate-chitosan aerogel fibers. The aerogel was not loaded with bioactive substances, so only the effects of alginate and chitosan were addressed. The aerogel was found to be non-cytotoxic, and it demonstrated antibacterial activity against *S. aureus* and *K. pneumoniae.* Moreover, the aerogel showed comparable results compared to a commercialized medical device used for wound healing, making it a promising alternative in treating wounds [52]. The effect of a novel chitosan/polyvinyl-alcohol/polycaprolactone aerogel loaded with curcumin was investigated in diabetic wounds. Animal studies proved that the aerogel created had wound-healing properties by absorbing excessive wound exudate. Additionally, it had an antibacterial effect, which reduced inflammation and promoted faster diabetic wound healing [104]. For diabetic wound healing, nanofiber aerogels modified with LL-37-mimic peptide W379 have also demonstrated their therapeutic effect in a recent study [105]. W379 peptide holds antibacterial properties [106], and it promoted cell infiltration, neovascularization, and re-epithelialization in diabetic wounds in in vivo and in vitro experiments. The mechanism behind wound healing involves the upregulation of the phospho-extracellular signal-regulated kinase (p38 MAPK) signaling pathway by W379, which results in re-epithelialization. The potential of aerogel-based scaffolds has also been studied. It involved photo-crosslinkable methacrylated silk fibroin (SF-MA) biopolymer and methacrylated hollow mesoporous silica microcapsules (HMSC-MA). The created scaffold exhibited antibacterial properties, facilitated by the sustained release of ciprofloxacin from HMSC-MA, which inhibited bacterial growth and biofilm formation. The porous structure of SF-MA aerogels supported cell infiltration, adhesion, and proliferation [107].

### 6.2. Aerogels in Drug Delivery

**Figure 5:**
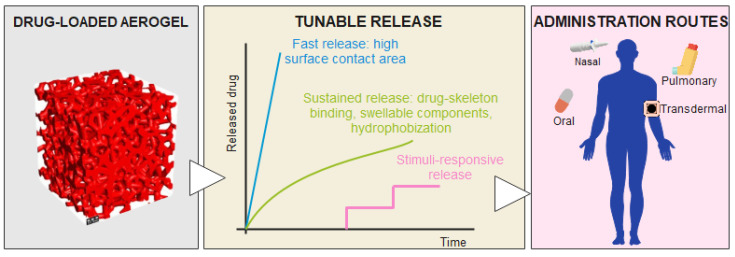
Aerogels in drug delivery. Adapted from an open-access source [95].

Aerogels are emerging as promising platforms for drug delivery systems since their unique properties make them ideal candidates for encapsulating and delivering therapeutic agents to specific sites within the body. They can be administered through different routes, for example, through nasal, oral, transdermal, or pulmonary administration. A schematic illustration of aerogel use in drug delivery is presented in Figure 5 [95].

Several studies have demonstrated that aerogels could improve the bioavailability of low-solubility drugs and their stability when loaded within the matrix. Qin et al. evaluated in vitro the release kinetics and characteristics of a cellulose aerogel loaded with resveratrol [108]. Under simulated gastric acidic environments, the drug was not degraded, demonstrating its stability when incorporated into the aerogel matrix. Cui et al. investigated the stability and effect of resveratrol-loaded TEMPO-oxidized cellulose aerogels in vivo [109]. First, when investigating the thermal stability, the drug-loaded aerogel’s decomposition temperature was higher than that of the cellulose aerogel but lower than that of pure resveratrol, which suggests that the aerogel matrix improves the thermal stability of resveratrol. The stability under gastric acidic fluidic conditions was also investigated. Pure resveratrol showed a much higher release rate than the loaded aerogel, indicating that the aerogel matrix provides a protective environment for resveratrol and improves its stability. Xie et al. created a mesoporous silica aerogel loaded with different antibacterial agents [79]. The evaluation of the release profile showed that the initial burst release followed by a controlled release profile demonstrates that the aerogel matrix can protect the antibacterial drugs and maintain their stability over an extended period. This effect also contributed to the hydrophobic nature and high porosity of the aerogel, along with the crystallization behavior of the antibacterial agents. Horvat et al. discovered that the addition of polylactic acid (PLA) to pectin aerogels loaded with two different drugs can improve their stability [110]. PLA improved the stability of the aerogels, extending their stability in simulated body fluid (SBF) from 24 h to more than one week, depending on the amount of PLA. The dissolution of both drugs was prolonged for up to 2 days, indicating that the aerogel matrix improves the drug’s stability and results in a controlled release.

The storage conditions for drug-loaded aerogels have also been studied. Zarinwall et al. [111] investigated the stability of ibuprofen-loaded silica aerogels in the drug. They discovered that the formulations containing 100% amorphized ibuprofen maintained their amorphous state without recrystallizing over a storage period of 6 months under ambient conditions. The loading of drug molecules inside the porous matrix of the aerogel inhibited recrystallization. The increased dissolution rates and the ability to prolong the release of ibuprofen through surface functionalization suggested that the aerogel matrix stabilizes the drug and improves its bioavailability and release profile. Another study investigated the drug stability when incorporated into an alginate aerogel over a period of 6 months [112]. During this time, the aerogels were subjected to various conditions like UV exposure, temperature drops, and shaking. It was discovered that the aerogel was able to protect the amorphous drug since its stability was not affected. In another study, it was also discovered that the aerogel is able to protect the drug during storage. However, the drug loading method can impact the drug’s stability and release kinetics after storage [113]. So, it is important to find a proper loading method and optimal drug concentration to maintain good storage stability.

Recently, research has focused on studying the effects of antimicrobial drug-loaded aerogels on medicine applications. In their work to create a drug delivery system, Afrashi et al. coated silica aerogels with crosslinked polyvinyl alcohol (PVA) nanofibers. This technology was designed to provide regulated local drug release for medical use. Because of their large surface area, the silica aerogels showed a faster drug release rate than pure fluconazole, suggesting they could be used as drug carriers. Adding PVA nanofibers to the aerogels and creating a composite material further regulated the drug release. The hydrophobicity of silica aerogel particles was a major factor in deciding how the system released drugs. The research proves aerogels can be a promising substrate for controlled drug delivery applications [114].

The capacity of starch aerogels crosslinked with trisodium citrate to release the volatile antifungal compound trans-2-hexenal was also studied. The medication was introduced into the aerogels by submerging them in an aqueous solution containing the chemical, and then Span 80 was sprayed onto their surface. According to the results, trans-2-hexenal release from the aerogel formulations significantly slowed down compared to the free compound. Furthermore, the aerogels’ surface coating further postponed the antifungal activity against *Aspergillus parasiticus*, taking 19 h to completely inactivate the fungus, compared to 14 h for the uncoated aerogel [115]. Vancomycin hydrochloride (Vancomycin HCl)-loaded chitosan aerogel microparticles provide a regulated and effective drug delivery method. Vancomycin HCl’s active form is efficiently preserved by these aerogels, and its controlled release ensures that the medication works as intended. The exact dosage control made possible by the polymeric matrix of chitosan aerogels minimizes the possibility of negative effects, in contrast to the direct use of drug powder. Vancomycin’s quick antimicrobial effect was highlighted by in vitro research that showed it suppressed *S. aureus* bacterial growth within 6 h of treatment. Vancomycin’s continuous release kinetics from the chitosan aerogels guarantee prolonged therapeutic concentrations, improving the effectiveness of treatment. This novel method is a useful supplement to antimicrobial therapy since it has the potential to treat bacterial infections more precisely and safely [116]. Wu. et al. investigated the antimicrobial potential of antibiotics and Cu-loaded alginate aerogel [117]. The findings demonstrated the sustained and prolonged release of the Cu ions, as well as strong antibacterial activity over several days in in vitro studies. The study highlights the potential of aerogel-based antimicrobial approaches, opening up possibilities for the development of new treatments for infections. Qin et al. studied the properties and release of resveratrol-loaded silica aerogel [118]. It was demonstrated that the synthesized aerogel demonstrated prolonged and controlled release. In vitro studies further showed the non-cytotoxic properties of the aerogel. The cell viability remained high when exposed to the aerogel, highlighting its biocompatibility. This investigation underscores the versatility and safety of the aerogel-based delivery system, offering a promising alternative for controlled drug release and therapeutic interventions. Xie et al. developed mesoporous silica aerogels loaded with three different antibacterial agents [79]. In vitro experiments showed that the aerogel was biocompatible, and the drug was released in a controlled and prolonged manner. Furthermore, it demonstrated antibacterial activity, inhibiting 99.99% of *E. coli.* Uddin et al. investigated the use of aerogels made from aqueous dispersions of anionic and CNFs as solid supports for enzymes and AgNPs, as well as their antibacterial effects [63]. Lysozyme-loaded CNF aerogels had moderate antimicrobial effects against both gram-positive (*S. aureus*) and gram-negative (*E. coli*) bacteria. However, stronger antibacterial activity was observed when loading it with AgNPs. The impact of storage conditions was also investigated in this study. It was found that cold and dry storage preserves the enzymatic and antibacterial activities of CNF aerogels better than room temperature or very humid environments.

### 6.3. Aerogels as Coatings

Coating medical implants is an important application to consider because it helps improve their performance and compatibility within the body. These coatings can enhance the implant’s durability, reduce the risk of infection, and promote better integration with surrounding tissues. Additionally, coatings can provide a controlled release of drugs or therapeutic agents, aiding in the healing process and minimizing complications post-surgery [119,120]. Biodegradable polysaccharide aerogel coatings containing xanthan and pectin were created for medical-grade stainless steel in a study. To address postoperative orthopedic applications and reduce pain, inflammation, and the possibility of material rejection, the study looked into the release of two non-steroidal anti-inflammatory medicines (NSAIDs) that were incorporated into the coatings. The aerogel coatings on the stainless-steel specimens showed regulated drug release profiles and localized and generalized corrosion resistance. Within a day, both NSAIDs were completely released, and in vitro tests using osteoblasts generated from human bone confirmed their biocompatibility [121]. A carbon platform coated with an aerogel was investigated for osteosarcoma treatment. Diclofenac sodium and indomethacin, two anti-inflammatory drugs, were incorporated into the aerogel coating. The findings demonstrated the controlled release of the drugs, while the surface improvement (extra roughness and larger surface area) stimulated the cells responsible for bone regeneration [122]. In a recent study, researchers have developed a hemostatic device that works under extreme hot or cold conditions. They created an asymmetric wetting nano-silica aerogel-coated gauze (AWNSA@G) with a layer-by-layer (LBL) structure. The in vitro and in vivo investigations demonstrated that the created coated-gauze was biocompatible and had stopped the bleeding in animal models faster, and the coagulation time was reduced [123].

Aerogels can also be used as coatings for textiles to achieve different functionalities, such as super-hydrophobicity (self-cleaning), flame protection, chemical protection, thermal insulation, or antimicrobial preservation [124]. Porous silica aerogel particles coated with polyurethane (PU) have improved the effectiveness of covering cotton textiles for chemical protection. Studies have demonstrated the superior breathability and chemical and water resistance of this type of material. No liquid flowed through the clothes since most of the chemicals were absorbed in the porous aerogel layer. As the concentration of aerogel particles in the PU-aerogel coating increased, there was a steady improvement in moisture vapor transfer and air permeability, which suggested enhanced thermal comfort [125]. These findings present new avenues for creating new textiles in which antimicrobial agents could be incorporated. New, improved textiles can be developed, ranging from medical gowns and PPE in healthcare settings to sportswear and home textiles, that could benefit from added protection against microbial growth.

## 7. Challenges and Future Perspectives

### 7.1. Overcoming Biocompatibility and Toxicity Challenges

Although aerogels are considered generally safe when choosing appropriate material, taking precautions before using them in healthcare or other areas is still necessary. The toxicity of nineteen organic and inorganic aerogels was investigated by Keller et al. They were studied for their stability, dissolving rate, and effect on cells; no substantial adverse effects were found. Polyurethane aerogel was considered a suitable test material for further evaluating its bioactivity in vivo. Very little cell damage was noticed, and it was concluded that it might have been reversible [125]. As mentioned earlier, a few studies demonstrated toxicity concerns, especially due to the aerogel’s accumulations in organs such as the lungs, kidneys, and liver [55,56].

Additional research is essential to comprehensively assess the safety of aerogels, given the current lack of data regarding their potential toxicity and biocompatibility. Most of the present studies evaluate the safety of silica aerogels [126], so in the future, it is essential to include other aerogels created from different materials to gain a deeper understanding.

### 7.2. Future Research Directions and Emerging Innovations

Over the years, there have been advances in creating new aerogels with unique properties and employing new synthesis methods. Eco-friendly synthesis of the aerogel precursors is an interesting method that could be studied more. This synthesis method involves agricultural, municipal, and industrial waste that could be converted into aerogels [127]. Waste offers great advantages, such as its high abundance and low cost, so it represents a great alternative for producing aerogels. However, it has some drawbacks, such as the complexity of the waste composition and the difficulty of large-scale production. It is expected that improvements in environmentally friendly recycling techniques will expand the types of waste materials that can be turned into aerogels in the future. This innovative approach also addresses the growing problem of waste accumulation. A more circular economy will be possible as research advances and recycling process optimization contributes to making waste resources more efficiently used [128].

Considering the applications for antimicrobial activity, the powerful antibacterial effects of plant extracts and essential oils have been the subject of several investigations despite a lack of research on their incorporation into aerogels. Numerous studies have shown that extracts from various plants, including alcoholic and aquatic sources, as well as essential oils, have strong antimicrobial action. Besides, their natural origin makes them biocompatible without causing harmful effects to the body [129,130]. Even with these encouraging statistics, more research is necessary to determine whether or not to incorporate them into aerogels, as the toxicity needs to be studied. Using plant extracts and essential oils in aerogel matrices to enhance their antibacterial properties may provide new approaches to combating bacterial infections in many contexts.

## 8. Conclusions

In conclusion, aerogels are a promising emerging field in antimicrobial therapy because of their unique properties, which make them excellent options for treating infections. This paper addresses the challenges of using aerogels while highlighting their importance in providing potent antibacterial treatments. Aerogels can be made from numerous materials, some of which are naturally occurring (such as chitosan), offering great versatility and availability, as well as synthetic ones. They can be loaded with therapeutic agents and can further be used for the desired applications. Aerogels provide an adaptable framework for the targeted and effective delivery of antimicrobial agents due to their remarkable surface area, porosity, and biocompatibility. Even with these advancements, issues like toxicity, biocompatibility, and the requirement for improved drug loading and release mechanisms need to be further addressed, as a gap in knowledge still exists. These challenges can be addressed with continued study and innovative approaches, opening the door to novel antimicrobial therapy uses and treatments. Prospective research directions involve studying novel materials, optimizing synthesis techniques, and maximizing the capabilities of natural antimicrobial agents. Aerogels have the potential to improve antimicrobial therapy by overcoming AMR, improving the antimicrobial agent’s stability, and providing controlled release, which could enhance global healthcare outcomes by implementing these innovations.

## Figures and Tables

**Table 1:** Aerogels classification [12].

Class	Examples
Organic	Polymer aerogels (Cellulose, Conducting polymers) Carbon aerogels (Carbon, Carbon nanotubes, Graphene)
Inorganic	Oxide aerogels (TiO_2_, SiO_2_, Al_2_O_3_, ZrO_2_, SnO_2_) Metallic and chalcogenide aerogels (Fe, Co, Ni, Pd, Pt, CdS, CdSe)
Composites	Mixed metal oxide aerogels (SiO_2_-TiO_2_, TiO_2_-ZnO) Other (Metal oxide aerogel composites, Carbon-based aerogel composites)

**Table 2:** Polymer-based aerogels and their advantages.

Material	Advantages	Ref.
Chitosan	- Antibacterial and antifungal properties - Wound healing acceleration - Hemostatic agent - Mucoadhesive	[27]
Alginate	- Strong antibacterial effect (especially against *S. aureus* and *E. coli*) - Biofilm inhibition—can disrupt and prevent the formation of microbial biofilms - Hemostatic properties	[28]
Cellulose	- High water absorption capacity - High and long-term antimicrobial activity - Wound healing support—by providing a conducive environment for tissue regeneration	[29]
Starch	- Non-toxic - Biodegradable - Can be loaded with antimicrobial agents	[30]

**Table 3:** Comparison between aerogels and conventional drugs in antimicrobial applications.

Feature	Aerogel-Based Antimicrobials	Traditional Antiseptics/Drugs
Stability	Good stability with sustained release [79,91]	Variable stability [92,93]
Drug resistance	No resistance reported	High AMR [94]
Dosage frequency	Reduced due to controlled release [95]	Frequent administration needed [96]
Side effects	Potentially reduced, targeted delivery [87]	Higher risk, affects a broader range of tissues [88]
Surface area	Large surface area due to pores [90]	Reduced, compared to aerogels [9,97]

## Data Availability

Not applicable.
